# The nature and distribution of road sediment contaminants in the greater Las Vegas, Nevada area

**DOI:** 10.1007/s10661-025-14725-9

**Published:** 2025-12-03

**Authors:** Gokey K. J., Gillis M., Brown K. L., Renkes N., McLeod C. L., Krekeler M. P. S.

**Affiliations:** 1 Department of Geology and Environmental Earth Science, Miami University, Oxford, OH 45056 USA; 2 Department of Geology and Environmental Geoscience, DePauw University, Greencastle, IN 46135 USA; 3 Department of Geoscience, University of Nevada, Las Vegas, Las Vegas, NV 89154 USA; 4 Department of Mathematical and Physical Sciences, Miami University, Hamilton, Hamilton, OH 45011 USA

**Keywords:** Road sediment, Urban pollution, Heavy metals, Anthropogenic spherules

## Abstract

Road sediment is an underutilized medium in the investigation of environmental pollution, yet it serves as both a source and sink for a wide array of pollutants. In urban areas especially, contaminants present within road sediment have the potential to pose human health risks depending on the abundance, chemical, and physical nature of these contaminants as well as the duration (i.e. acute or chronic) of exposure. Las Vegas, Nevada is currently one of the fastest growing urban areas in the United States and is therefore a prime location for the investigation of potential environmental contaminants and pollutants in road sediment. Forty-six road sediment samples were collected from locations throughout the Las Vegas region, including Las Vegas, Henderson, and Boulder City. Geochemical and mineralogical characterization of the selected samples was completed using scanning electron microscopy (SEM), transmission electron microscopy (TEM), and X-ray fluorescence spectrometry (XRF). Results from these analyses indicate the presence of metal-bearing particulate matter (including metal shavings), in addition to spherules, all of which are consistent with an anthropogenic origin. Additionally, geoaccumulation index (*I*_*geo*_) values determined from XRF bulk chemical data show that Cu and Zn were enriched in road sediment samples (*I*_*geo*_ > 1) throughout Las Vegas and are thus considered to be significant anthropogenic pollutants in the region. This study serves as the first investigation into road sediment contaminants in the region, and provides a critical framework for more detailed investigation of the source and potential human health effects of these contaminants.

**Supplementary Information:**

The online version contains supplementary material available at 10.1007/s10661-025-14725-9.

## Introduction

Road sediment is a heterogeneous mixture of geogenic, anthropogenic, and organic material deposited in or along a road, and it is present within rural, urban, or suburban communities (Andrews & Sutherland, 2004; Dietrich et al., 2019, 2022a; Flett et al., 2016; Khan & Strand, 2018; Teran et al., 2020). It is an easily accessible medium which often reflects the natural and anthropogenic activity of the local environment (Abdelaal et al., 2021; Dietrich et al., 2018, 2019, 2022a; Flett et al., 2016; O’Shea et al., 2020), and therefore serves as both a source and sink for environmental contaminants (e.g., heavy metals and metalloids; Buck et al., 2013; Dietrich et al., 2019, 2022a; Khan & Strand, 2018; LeGalley & Krekeler, 2013; Li et al., 2013; Loganathan et al., 2013; Men et al., 2018; O’Shea et al., 2020; Wiseman et al., 2018, 2021; Zgłobicki et al., 2018). In this contribution, the definitions of contaminant and pollutant outlined in Chapman (2007) are utilized. Here, a contaminant may be defined as any unwanted substance (e.g., chemical or biological) that is present in a location where it should not be or in concentrations above natural background values. Additionally, a pollutant is defined as a contaminant that causes or may have the potential to cause adverse impacts to the environment and human health. Based upon these definitions, all pollutants are contaminants, but not all contaminants are pollutants.

Despite the accessibility of road sediment, it has been historically understudied in the United States (US) compared to other regions globally (i.e., Asia, Europe, Australia; Amato et al., 2009; Charlesworth et al., 2003; Dietrich et al., 2022a; Li et al., 2013; Liu et al., 2016; Men et al., 2018). Furthermore, there have been few studies of road sediment in arid and semi-arid environments in the U.S.; instead, most previous studies have focused on relatively temperate (e.g., Ohio, Pennsylvania) and tropical regions (e.g., Hawaii; Allen et al., 2024; Andrews & Sutherland, 2004; Dietrich et al., 2018, 2019; Flett et al., 2016; LeGalley & Krekeler, 2013; LeGalley et al., 2013; O’Shea et al., 2021a; Sutherland, 2003).

Las Vegas, Nevada, is an ideal setting for road sediment contaminant analysis as it is located in a semi-arid environment, receiving < 11 cm of total annual rainfall (Joshi et al., 2020; Thakali et al., 2016). No prior studies of road sediment in the region have been conducted. In arid and semi-arid environments, anthropogenic and wind activity may serve as the primary mode of remobilization for road sediment contaminants as compared to water in more humid environments (Buck et al., 2011, 2013, 2016; Goossens et al., 2012; Reheis & Kihl, 1995; Reheis & Urban, 2011; Soukup et al., 2012). Owing to the drier conditions in the region, the textural and chemical properties of road sediment in arid/semi-arid environments may have fundamentally different textural and chemical properties from those found in more humid environments. Furthermore, the introduction of road sediment into the atmosphere by wind activity and anthropogenic reworking (e.g., street sweeping) may facilitate inhalation or ingestion of potential contaminants, especially those associated with finer size fractions (< 10 µm), which may present a potential human health risk that is ultimately dependent on the chemical and physical nature of contaminants present (Fubini & Fenoglio, 2007; Laidlaw & Taylor, 2011; Buck et al., 2013; Wiseman & Zereini, 2014; Goossens et al., 2015; Buck et al., 2016; Yang et al., 2016; Keil et al., 2018; Wiseman et al., 2021; US EPA, 2024).

The city of Las Vegas is located in the Las Vegas Valley, Clark County, Nevada. This valley is a ~ 4,100 km^2^ fault-bounded structural basin located within the Basin and Range physiographic province (Thakali et al., 2016; Wernicke et al., 1988). Las Vegas is currently one of the fastest-growing cities in the US, boasting a population of > 2 million people for the entirety of the Las Vegas Metropolitan Statistical Area (Deacon et al., 2007; Joshi et al., 2020) and having grown from a population of ~ 1.4 M to ~ 2.3 M between 2000 and 2022 (USGS, 2024). Previous road sediment studies have documented the increased risk of environmental contaminant exposure in urban areas (Amato et al., 2009; Apeagyei et al., 2011; Dietrich et al., 2022b; Filippelli et al., 2005, 2015, 2018; Haynes et al., 2020; Hwang et al., 2016; Khan & Strand, 2018; Yang et al., 2023). In an area like Las Vegas where road sediment and dust emissions in the atmosphere may be exacerbated by wind or other anthropogenic activities (Amato et al., 2009; Buck et al., 2013; Goossens et al., 2012, 2015; Laidlaw et al., 2012; Padoan et al., 2017; Reheis & Kihl, 1995; Reheis & Urban, 2011; Soukup et al., 2012), large populations may be exposed to a variety of road sediment contaminants which may cause deleterious health effects in humans (Loganathan et al., 2013; DeWitt et al., 2017; Keil et al., 2018; Haynes et al., 2020; Dietrich et al., 2022b; US EPA, 2024).

The severity of the health risk from road sediment depends on both the chemical and physical properties of the contaminants present (Laidlaw et al., 2005; Fubini & Fenoglio, 2007; Laidlaw & Filippeli, 2008; Amato et al., 2009; Irvine et al., 2009; Loganathan et al., 2013; Wiseman et al., 2014; Yang et al., 2016; DeWitt et al., 2017; Khan & Strand, 2018; Wiseman et al., 2018; Keil et al., 2018; Haynes et al., 2020; Wiseman et al., 2021). Potential road sediment pollutants in Las Vegas may include heavy metals and metalloids known to be detrimental to human health such as Pb, Zn, Cr, Cu, Mn, As, Ni (Laidlaw et al., 2005; Fubini & Fenoglio, 2007; Amato et al., 2009; Laidlaw & Taylor, 2011; Filippelli et al., 2015; Buck et al., 2016; DeWitt et al., 2017; Filippelli et al., 2018; Khan & Strand, 2018; Keil et al., 2018; Dietrich et al., 2019; Dietrich et al., 2022b), anthropogenic spherules, and a myriad of other anthropogenically derived contaminants (e.g., metal shavings, paint, plastics), all of which have been observed in previous road sediment studies (Sutherland, 2003; LeGalley et al., 2013; LeGalley & Krekeler, 2013; Flett et al., 2016; Padoan et al., 2017; Dietrich et al., 2018; Khan & Strand, 2018; Zgłobicki et al., 2019; O’Shea et al., 2020; Teran et al., 2020; O’Shea et al., 2021a; Dietrich et al., 2022a; Allen et al., 2024). While the human population of Las Vegas may be exposed to these potential contaminants by means of ingestion, inhalation, or dermal contact, of these three exposure pathways, ingestion and inhalation are the most rapid methods of uptake (Laidlaw & Filippelli, 2008; Wiseman & Zereini, 2014; Laidlaw et al., 2017; Men et al., 2018; Dietrich et al., 2022b; US EPA, 2024). Ingestion of contaminants may occur via contaminated food and water or hand-to-mouth activity. Inhalation of particulate matter (PM) may also occur, with PM size fractions < 10 µm in diameter being the most detrimental to human health (Amato et al., 2009; Wiseman et al., 2018; Wiseman & Zereini, 2014; Padoan et al., 2017; Khan & Strand, 2018; Wiseman et al., 2021; US EPA, 2024).

Overall, the availability of road sediment to serve as a source and sink of a wide array of contaminants contributed from a large area makes source identification challenging, and the lack of study of road sediment in arid environments makes Las Vegas a prime location for a detailed investigation. Additionally, the road sediment contaminants may fundamentally differ in their textural and chemical nature in arid environments as compared to more humid climates. Thus, geochemical and mineralogical characterization of sediment is imperative to address the gap in arid and western US road sediment literature, as well as provide further insight into pollutants in the region to better assess potential environmental and human health risk and provide suggestions for future road sediment investigation in the region and throughout arid environments globally.

## Methods

### Sampling

**Fig. 1:**
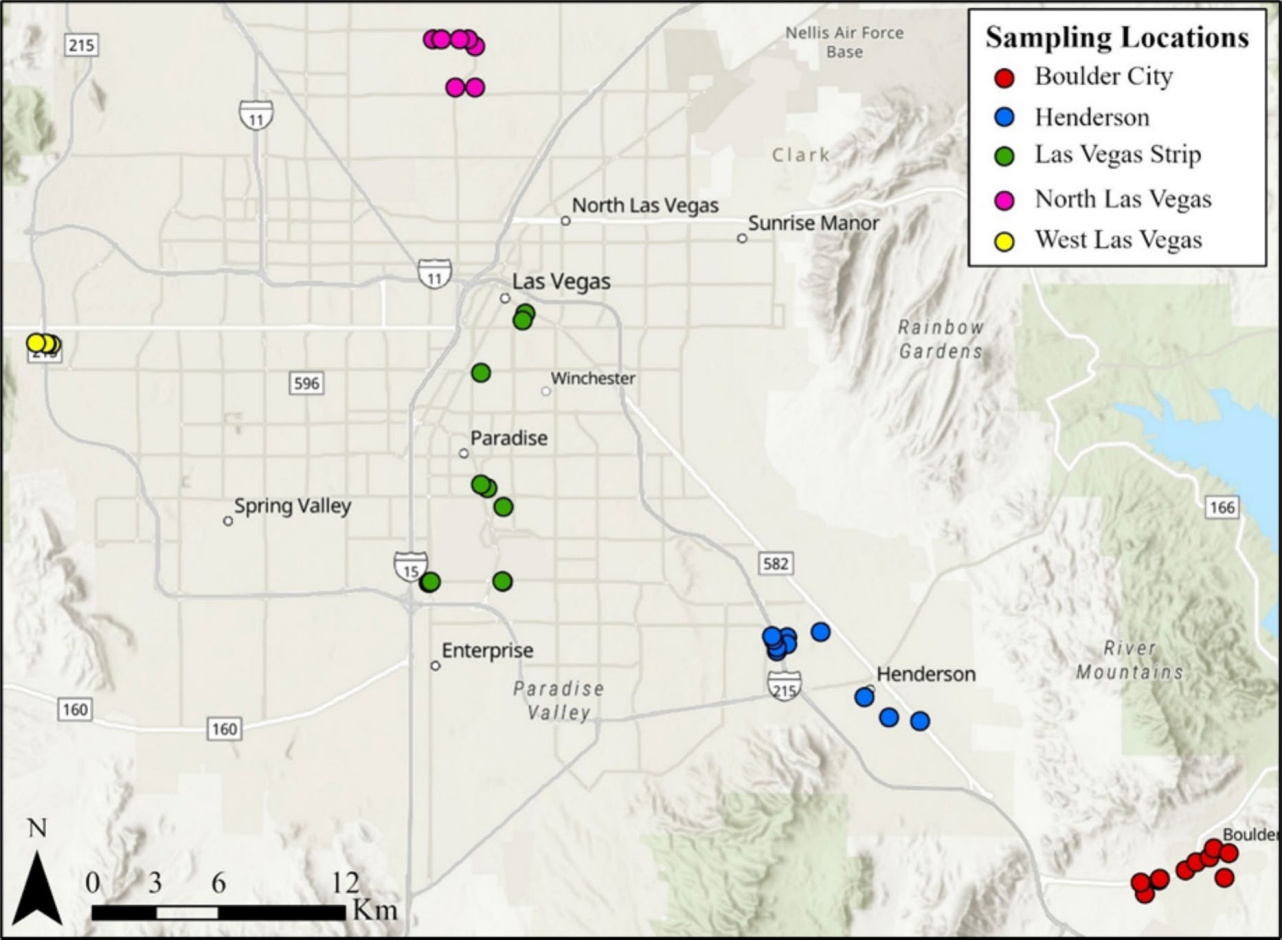
Map for all road sediment sampling sites in Boulder City (red), Henderson (blue), Las Vegas Strip (green), North Las Vegas (pink), and West Las Vegas (yellow). GPS coordinates for all sample sites are given in Supplemental Figures Table 3, Scanning electron microscopy (SEM)

Forty-six road sediment samples were collected near road curbs in Las Vegas, Henderson, and Boulder City, Nevada, over the course of several days during the COVID-19 pandemic (September 2020; Fig. 1, Supplemental Figures, Table 1). Ten were collected from North Las Vegas (NLV), ten from Boulder City (BC), ten from Henderson (H), twelve from the Las Vegas Strip (LVS), and four from West Las Vegas (WLV). Sampling locations included locations associated with the following: dual-triple-lane highways, dual-triple-lane highway 4-way intersections, residential areas and associated parking lots, business-dominated areas and associated parking lots, areas along roads with and without painted markings, roads beneath bridges, roads adjacent to urban-dominated landscapes, roads adjacent to desert-dominated landscapes (± vegetation cover), roads adjacent to community parks, roads paralleled by sidewalks, and those with and without medians (variably vegetated). All 46 road sediment samples were collected using plastic spoons to scoop street sediment into clear plastic sampling bags, and each sample was derived from within a < 0.05m^2^ sampling area. Plastic collection utensils were used to avoid metal contamination. New plastic spoons were used at each sampling location to prevent cross-contamination. Following shipping, all 46 samples were dried in an oven at 52 °C for 24 h to remove moisture. Following drying, samples were sieved using a 500 µm brass sieve and placed in separate sample bags based on their size fractions. This was done to reduce sample heterogeneity by removing large gravel and vegetation debris.

**Table 1:** Average bulk XRF analyses (*n* = 5 replicates) for nine elements in each road sediment sample with Rudnick & Gao (2003) average upper continental crust (UCC) concentrations and Smith et al. (2013) background regional topsoil concentrations for comparison

Sample #	V^a^	Cr^a^	Ni^a^	Cu^a^	Zn^a^	As^a^	Pb^a^	TiO_2_^b^	Fe_2_O_3_^b^	Sample #	V^a^	Cr^a^	Ni^a^	Cu^a^	Zn^a^	As^a^	Pb^a^	TiO_2b_	Fe_2_O_3b_
BC1	22	BDL	16	40	121	5	21	0.23	2.90	LVS3	90	BDL	14	457	1204	6	BDL	0.35	2.73
BC2	123	38	22	56	129	9	26	0.52	4.86	LVS4	97	56	14	376	1265	7	BDL	0.35	3.28
BC3	69	36	15	40	98	9	25	0.39	3.82	LVS5	87	BDL	13	154	1056	10	BDL	0.27	2.58
BC4	50	BDL	14	38	127	8	54	0.34	3.51	LVS6	68	BDL	10	162	689	6	BDL	0.23	2.37
BC5	67	29	14	34	192	13	39	0.29	4.09	LVS7	64	BDL	11	70	414	10	20	0.28	1.95
BC6	39	BDL	10	84	710	9	24	0.21	3.35	LVS8	88	BDL	13	137	472	15	69	0.37	2.49
BC7	BDL	41	20	108	597	14	37	0.16	4.13	LVS9	38	BDL	9	56	326	12	BDL	0.11	1.20
BC8	25	BDL	18	63	286	9	70	0.24	3.04	LVS10	60	22	BDL	33	243	7	118	0.16	1.29
BC9	88	BDL	20	93	627	10	39	0.34	3.23	LVS11	44	BDL	11	44	198	10	95	0.19	1.75
BC10	BDL	31	18	178	247	29	28	0.06	5.82	LVS12	45	BDL	19	71	321	4	25	0.17	1.33
H1	125	27	26	270	254	8	27	0.50	4.55	NLV1	37	BDL	BDL	98	123	7	BDL	0.16	1.66
H2	120	BDL	14	48	232	7	24	0.52	4.09	NLV2	58	BDL	12	115	629	10	BDL	0.19	2.17
H3	93	BDL	BDL	24	160	11	49	0.43	5.00	NLV3	47	BDL	BDL	41	224	5	BDL	0.20	1.72
H4	82	59	17	33	104	8	25	0.44	4.20	NLV4	57	BDL	14	44	142	5	BDL	0.18	1.64
H5	53	BDL	11	65	368	8	BDL	0.31	2.94	NLV5	210	BDL	10	58	220	6	32	0.95	2.20
H6	103	24	24	298	1537	9	BDL	0.43	3.40	NLV6	82	BDL	11	54	488	12	23	0.31	2.08
H7	76	49	20	393	961	9	BDL	0.30	3.49	NLV7	53	BDL	12	79	238	8	16	0.23	2.34
H8	78	36	20	117	990	10	33	0.35	3.51	NLV8	51	BDL	11	72	302	8	BDL	0.28	2.33
H9	48	45	15	143	1096	12	BDL	0.36	4.08	NLV9	35	31	26	68	383	10	34	0.23	4.71
H10	64	49	19	86	578	9	23	0.35	3.34	NLV10	40	BDL	10	40	270	7	BDL	0.14	1.92
WLV1	40	BDL	BDL	35	106	8	22	0.14	2.05	Min	22	22	9	24	98	4	16	0.06	0.79
WLV2	82	BDL	15	119	150	BDL	18	0.24	0.79	Max	210	59	26	546	2199	29	118	0.95	6.20
WLV3	46	31	14	138	597	13	BDL	0.25	3.30	St. Dev	33	10	5	118	438	5	24	0.14	1.22
WLV4	102	35	13	546	2199	27	BDL	0.32	6.20	Rudnick & Gao, 2003 (ppm)	97	92	47	28	67	5	17	0.64	
LVS1	60	BDL	22	126	517	12	BDL	0.20	2.44	Smith et al., 2013 (ppm)	44	22	13	14	54	7	20	0.25	
LVS2	89	40	13	244	948	7	BDL	0.36	2.98										

^a^Concentration in parts per million (ppm); ^b^Concentration in wt%

Twenty-nine samples were selected for comprehensive characterization via SEM. The finest sample fraction (< 500 µm) was selected for SEM analysis because it contains particles that are easily ingested or inhaled (US EPA, 2024). Additionally, the 29 samples selected for SEM represent a broad swath of road sediment locations within the Las Vegas region, as sediment samples from all five sampling regions were included for analysis. Selected sediment samples were mounted on aluminum scanning electron microscope (SEM) stubs via carbon adhesive tabs, and following initial mounting, were carbon coated to reduce electron charging during imaging.

SEM analyses were conducted using a Zeiss Supra 35 VP field emission scanning electron microscope (FE-SEM) at the Miami University Center for Advanced Microscopy and Imaging (CAMI). Analyses were completed using nitrogen (N_2_) as the compensating gas in variable pressure (VP) mode typically ranging from 25–90 Pa to help further mitigate electron charging. The SEM is equipped with a backscatter detector (BSD) and an energy dispersive spectrometer (EDS; EDAX2000). The elemental detection limit for EDS is approximately 0.1 wt% for most elements (e.g., Allen et al., 2024; Dietrich et al., 2019; Krekeler et al., 2010). Images were taken in BSD mode to better identify heavy elements within sample material. Following imaging, EDS analysis of sample material allowed for the acquisition of basic chemical composition data for particles of interest in road sediment samples. A combination of VP, high voltage (25 kV), and the BSD imaging mode improves the user’s ability to discriminate between particles with higher average atomic numbers and those with low atomic numbers, resulting in better identification of metal-rich particles (e.g., Ti, Cr, Fe, Ni, Cu, Zn, and Pb).

The SEM instrumentation and parameters described above have been used extensively in previous investigations of a wide variety of sample matrices (e.g., Krekeler et al., 2010; Barrett et al., 2011; Schellenbach & Krekeler, 2012; Varma Sinha et al., 2015; Barnes et al., 2020; Cymes et al., 2020; Klein & Krekeler, 2020; Lindeman et al., 2020; Oglesbee et al., 2020; Cymes et al., 2021; Flett et al., 2021; O’Shea et al., 2021b).

### Transmission electron microscopy (TEM)

Seventeen road sediment samples from all five sampling areas were selected for bright field transmission electron microscopy energy dispersive spectroscopy (BF-TEM-EDS). Selected samples were prepared via suspension by first placing a small amount (< 1 g) of < 50 µm road sediment into a glass vial and adding approximately 2 mL of 100% denatured ethanol. Suspensions were agitated for 5 min by hand and then allowed to sit for 1 min to allow larger particles to settle. The finest sediment suspension material was then pipetted from the vials and applied to 3.0 mm lacey carbon-supported copper grids. Sediment suspension concentrations varied depending on the primary makeup of the sample (e.g., samples with excess biological material needed more sediment added to the vials to produce satisfactory suspensions). Overall, these ethanol suspension preparations resulted in numerous particles deposited in a well-dispersed manner on the copper grids.

Bright field TEM analyses were acquired using a JEOL JEM 2100 TEM operated at 200 keV in bright field TEM mode at Miami University’s CAMI facility. The TEM is equipped with a LaB_6_ electron source, a JEOL Bright Field TEM detector, and a Bruker Quantax 200 STEM EDXS system, which were all used extensively for sample analysis. TEM images were collected for all particles observed with EDXS, and selected area electron diffraction (SAED) was also collected when the appropriate thickness and degree of crystallinity in the particle were met.

The FE-SEM and TEM-EDS preparation methods described above have been used extensively in previous mineralogical and sedimentological studies (Krekeler et al., 2010; Barrett et al., 2011; Buck et al., 2013; LeGalley & Krekeler, 2013; White et al., 2014; Cymes et al., 2017, 2020; Paul et al., 2017; Dietrich et al., 2018, 2019; Murchland et al., 2024; Jenkins et al., 2024).

### X-Ray fluorescence spectroscopy (XRF)

Elemental concentrations of major, minor, and trace elements of all 49 sediment samples were acquired using a Bruker TRACER 5G handheld p-XRF at DePauw University, Indiana. The p-XRF is equipped with a rhodium source (Rh), a graphene window, a silicon drift detector (SDD), and an 8 mm collimator. Sieved sample material (< 500 µm) was utilized for all 49 sediments to minimize the amount of gravel and vegetation within each sample as well as to create a uniform particle distribution. This approach is similar to that method outlined in Laperche & Lemière (2021).

All 46 road sediment samples were analyzed via p-XRF under atmospheric conditions. Each sample was analyzed a minimum of five times at 60 s per run (30 s at 45 kV, 30 s at 20 kV) using a Fundamental Parameters (FP) two-phase Heavy Metals and Nutrients in Soils/Sediment calibration. This p-XRF configuration is capable of producing characteristic X-rays of elements ranging from sodium (Na) to uranium (U). Analysis was completed by measuring approximately 4–5 g of 500 µm sediment material onto a 4.0 µm thick Prolene thin-film, which was then placed directly over the p-XRF analysis window. Prolene thin-film was replaced after the analysis of each sample to prevent cross-contamination between samples. Both accuracy and precision were maintained by analyzing standard materials JSd-1 and JSd-2 (Geochemical Survey of Japan Reference Materials; Imai et al., 1996), both of which had known elemental values. Bulk XRF concentrations for all street sediment samples and standards, JSd-1 and JSd-2, are reported in Supplemental Figures Table 2. Manufacturer-stated limits on detection (LOD) are Ti (0.022 wt%); V (2 ppm); Cr (5 ppm); Ni (< 5 ppm); Cu (< 5 ppm); Zn (< 5 ppm); As (< 5 ppm); Pb (< 5 ppm).

**Table 2:** Calculated average *I*_*geo*_ values using Smith et al. (2013) topsoil elemental concentrations near the Las Vegas region for selected elements for each sampling region of Las Vegas. The average *I*_*geo*_ value for each element for the entire Las Vegas Area is given, as well as the minimum and maximum *I*_*geo*_ values and standard deviation

	V	Cr	Ni	Cu	Zn	As	Pb
BC Average	−0.36	−0.94	−2.04	−0.06	1.86	−2.70	−0.97
H Average	0.27	−0.76	−1.89	0.61	2.75	−2.90	−1.20
WLV Average	−0.08	−1.01	−2.26	1.00	2.51	−2.22	−1.75
LVS Average	−0.01	−0.86	−2.36	0.83	3.00	−3.04	−0.35
NLV Average	−0.22	−1.13	−2.44	−0.08	2.01	−3.15	−1.41
Average Whole City	−0.08	−0.94	−2.20	0.46	2.43	−2.80	−1.14
Min	−1.63	−1.60	−2.97	−1.47	0.55	−4.06	−2.06
Max	1.66	−0.17	−1.37	3.04	5.05	−1.21	0.83
St Dev	0.64	0.39	0.42	1.12	1.17	0.54	0.72

### Geoaccumulation indices (I_geo_)

The geoaccumulation index (*I*_*geo*_) was originally introduced by Müller (1969) to measure contamination levels of heavy metals in stream sediments; however, this technique may also be utilized in the context of soils and sediments, including road sediment material (Yaqin et al., 2008; Barbieri, 2016; Trujillo-González et al., 2016; Dietrich et al., 2022a). The *I*_*geo*_ classification system is designed to rank each element from a given sample on a six-point class scale wherein *I*_*geo*_ ≤ 0 material = unpolluted; 0 ≤ *I*_*geo*_ ≤ 1 = unpolluted to moderately polluted; 1 ≤ *I*_*geo*_ ≤ 2 = moderately polluted; 2 ≤ *I*_*geo*_ ≤ 3 = moderately to strongly polluted; 3 ≤ *I*_*geo*_ ≤ 4 = strongly polluted; 4 ≤ *I*_*geo*_ ≤ 5 = strongly to extremely polluted; and *I*_*geo*_ > 6 material is extremely polluted (e.g., Abdullah et al., 2020; Haris et al., 2017; Looi et al., 2019).

Geoaccumulation index (*I*_*geo*_) values were calculated (Eq. 1) to determine the extent of major, minor, and trace element contamination of Las Vegas road sediment material.

1 $${I}_{geo}=log2\left(\frac{{C}_{n}}{{1.5B}_{n}}\right)$$

where *C*_*n*_ is the bulk XRF measured concentration of a given element within road sediment material and *B*_*n*_ is the concentration of a reference material. Bulk p-XRF elemental concentrations of the 46 Las Vegas road sediment samples were compared to average elemental concentrations for topsoil samples (*n* = 20; depths < 5 cm) from the Las Vegas region (from Smith et al., 2013). The average topsoil concentrations of selected heavy metals and metalloids (e.g., V, Cr, Ni, Cu, Zn, As, and Pb) were used to represent regional background concentrations (*B*_*n*_). Upper continental crust (UCC) elemental concentrations of selected metals and metalloids as determined in Rudnick & Gao (2003) were also utilized in calculating *I*_*geo*_ for the Las Vegas region. Although topsoil elemental concentrations from Smith et al. (2013) better reflect the regional background concentrations, the Rudnick & Gao (2003) *I*_*geo*_ values place the Las Vegas road sediment data into a broader global context. These data are presented in Supplemental Figures, Fig. 1 and Table 3. This approach for determining the geoaccumulation index for road sediments has been used in previous studies (Li et al., 2013; Flett et al., 2016; Dietrich et al., 2018).

**Table 3:** Calculated average *I*_*geo*_ values using Rudnick & Gao (2003) upper continental crust (UCC) and road sediment elemental concentrations near the Las Vegas region for selected elements for each sampling region of Las Vegas. The average *I*_*geo*_value for each element for the entire Las Vegas Area is given, as well as the minimum and maximum *I*_*geo*_ values and standard deviation

	V	Cr	Ni	Cu	Zn	As	Pb
BC Average	−1.49	−2	−2.12	0.6	1.27	0.51	0.41
H Average	−0.86	−1.81	−1.98	1.5	2.32	0.27	0.18
WLV Average	−1.21	−2.07	−2.34	1.66	1.92	0.99	−0.37
LVS Average	−1.14	−1.91	−2.44	1.49	2.41	0.17	1.03
NLV Average	−1.35	−2.18	−2.52	0.58	1.42	0.06	−0.03
Average Whole City	−1.21	−1.99	−2.28	1.17	1.87	0.4	0.25
Min.	−2.76	−2.65	−3.05	−0.81	−0.04	−0.85	−0.67
Max.	0.53	−1.23	−1.45	3.7	4.45	2	2.21
St. Dev.	0.64	0.39	0.42	1.12	1.17	0.54	0.72

## Results

### Scanning electron microscopy (SEM)

SEM–EDS techniques show several contaminants within road sediment samples that include heavy metals/metalloids, metal shavings, and anthropogenic spherules.

**Fig. 2:**
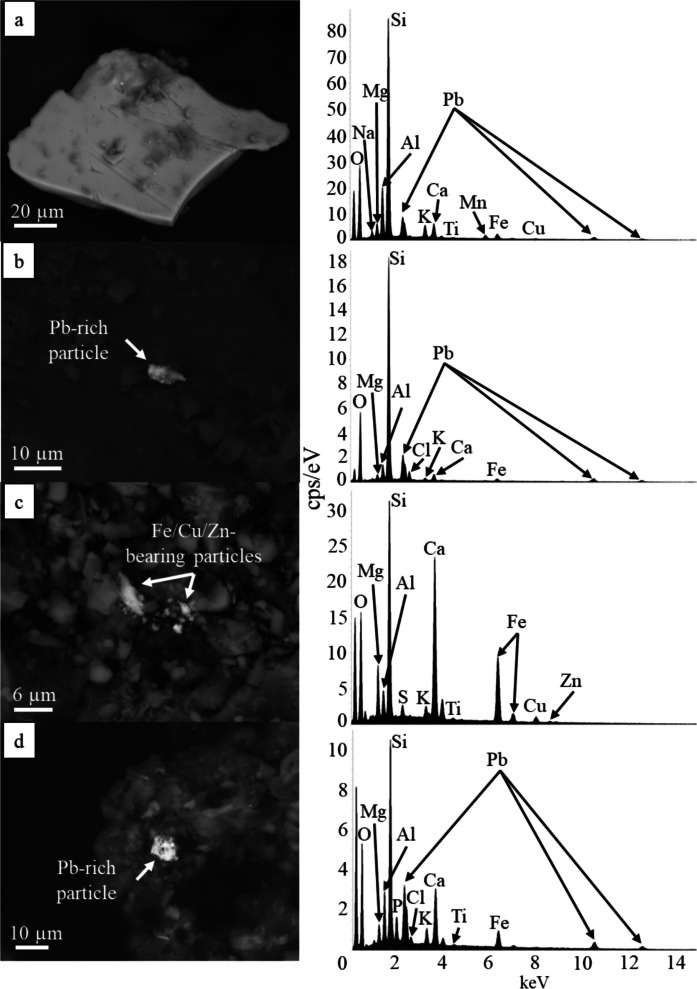
SEM BSD images and respective EDS spectra of road sediment particles containing heavy metals. **a**) Pb-rich particle containing minor Mn and Fe. **b**) Pb-rich particle surrounded by Si-rich detrital grains. **c**) Fe-rich particle bearing minor Cu and Zn surrounded by Si and Ca-rich detrital grains. **d**) Pb-rich particle surrounded by Si-rich detrital grains

Heavy metal and metalloid-bearing particles identified with SEM are typically anhedral in nature and vary in size from ~2 µm to >100 µm (Fig. 2). While most heavy metal-bearing particles occur as discrete grains (Fig. 2a-b), some of the smallest size fractions (<10 µm) occur as aggregates of multiple small particles (Fig. 2c-d). Energy dispersive spectroscopy (EDS) analyses reveal that these metal-bearing particles are rich in Fe, Cr, V, Cu, Ni, and Zn, with minor amounts of Pb.

**Fig. 3:**
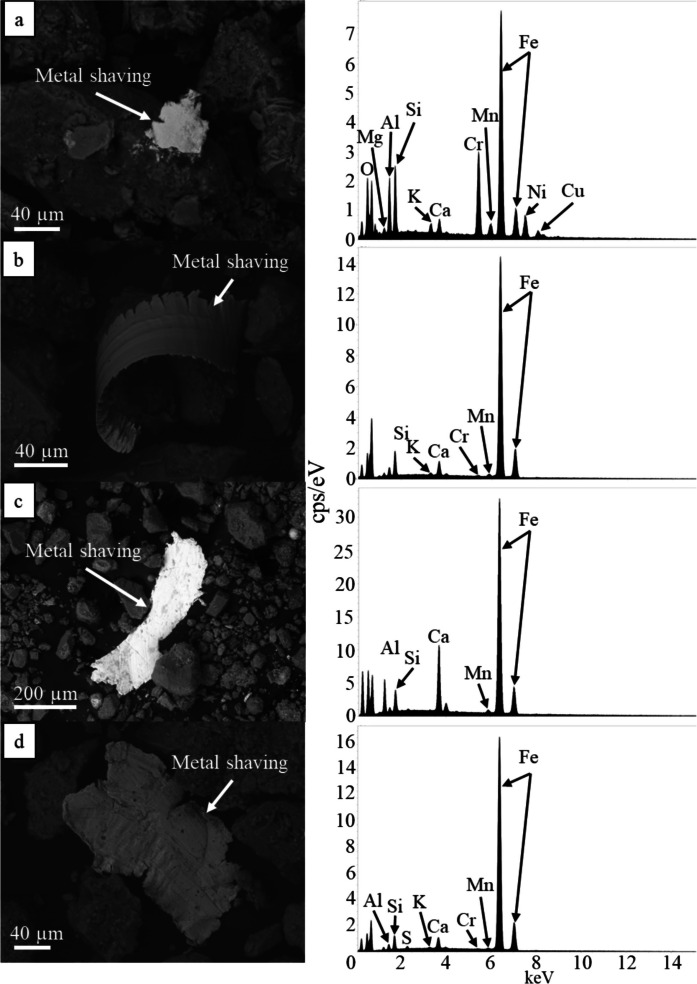
SEM BSD images and respective EDS spectra of anthropogenic metal shavings in Las Vegas road sediment. **a**) Fe-rich metal shaving bearing Cr, Mn, Ni, and Cu surrounded by Si-rich detrital grains. **b**) Fe-rich metal shaving bearing minor Mn and Cr surrounded by Si-rich detrital grains. **c**) Fe-rich metal shaving containing minor Mn. **d**) Fe-rich metal shaving containing minor Cr and Mn

In addition to heavy metals, anthropogenically derived metal shavings are also present in Las Vegas road sediment samples. These particles vary in size from < 40 µm to > 200 µm in length, and approximately 30 µm to 100 µm in width, with a range of 70 µm to 100 µm occurring most often (Fig. 3). Metal shaving textures show that they are generally anhedral with hackly fractures, jagged edges, and surface textures consistent with that of abrasion. Longer shavings (length > 100 µm) are curled, and their surfaces are typically smooth with grooves etched into them (Fig. 3b-c). Minor pitting is observed on the surfaces of most shavings, and pits are typically < 10 µm in diameter. All metal shavings identified with SEM are enriched in Fe and contain Mn, as indicated by minor EDS peaks (Fig. 3). Cr is frequently present in metal shaving EDS spectra (Fig. 3a, b, d). Ni and Cu are observed less frequently (Fig. 3a).

**Fig. 4:**
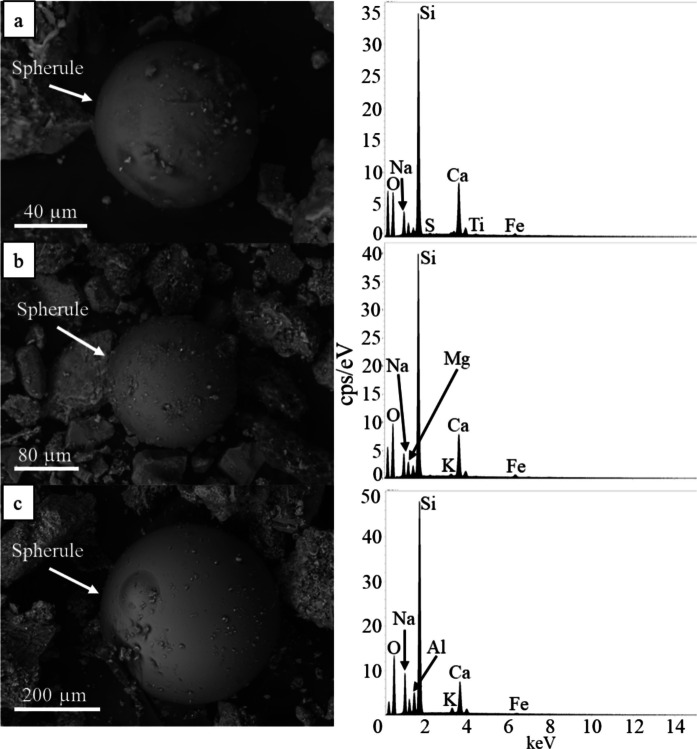
SEM BSD images with representative EDS spectra of glassy technogenic spherules identified in Las Vegas road sediment. Spherules are rich in Si and contain Na, Ca, and Fe. **a**) 80 μm spherule displaying minor pitting on its surface. **b**) Approximately 80 μm spherule. **c**) Approximately 400 μm spherule showing little evidence of mechanical abrasion

**Fig. 5:**
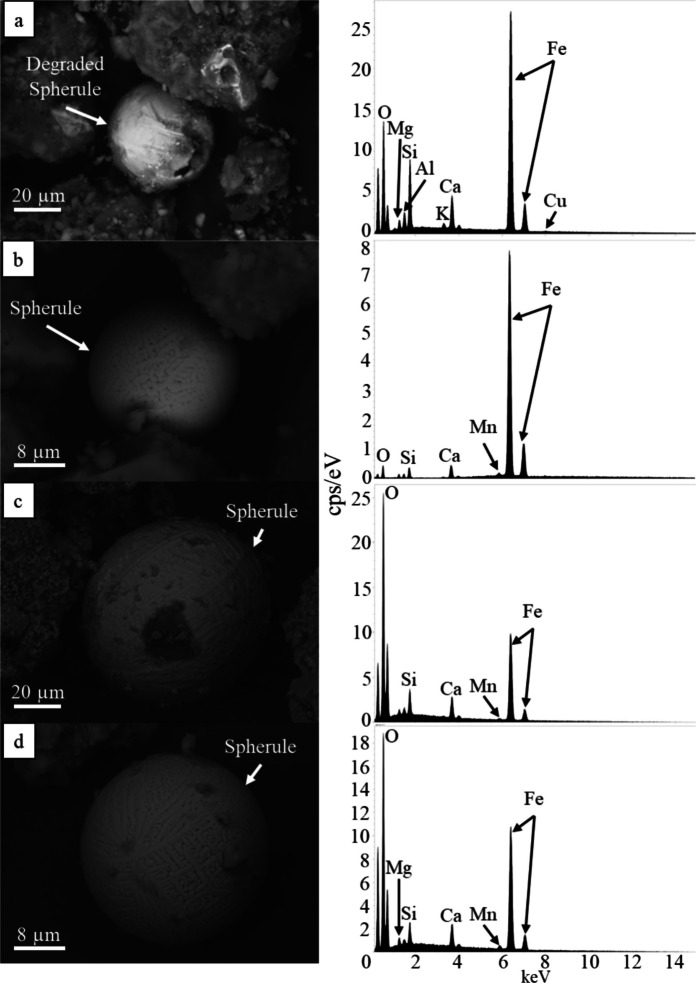
SEM BSD images with representative EDS spectra of metal-rich technogenic spherules identified in Las Vegas road sediment. All of these spherules are enriched in Fe and contain Si and Ca. **a**) Degraded glassy metal-bearing spherule approximately 30 μm in diameter. **b**) This spherule is approximately 20 μm in diameter with visible crystal boundaries. **c**) Approximately 70 μm diameter spherule displaying dendritic surface textures. **d**) Approximately 25 μm spherule displaying visible crystal boundaries between dendritic crystals and spinel textures

Anthropogenic spherules are observed in all 29 road sediment samples characterized via SEM–EDS. These spherules frequently vary in size from ~2 µm to >100 µm in diameter (Figs. 4 and 5). Two distinct populations of spherules are identified, with one being enriched in Si (Fig. 4) and the other enriched with Fe (Fig. 5). Si-rich spherules have smooth, glassy surface textures and commonly display conchoidal fractures and minor surface pitting (Fig. 4). These glassy spherules are primarily composed of Si and O with major amounts of Ca; they do not contain heavy metals above EDS detection limits (0.1 wt%; Fig. 4). Fe-rich spherules exhibit glassy surface textures (Fig. 5a), as well as dendritic and skeletal growth patterns (Fig. 5b-d). Those with dendritic patterns occur more often. All Fe-rich spherules are enriched with Fe and O and contain variable amounts of Si and Ca. All spherules with dendritic and skeletal growth patterns contain minor Mn (Fig. 5b-d); however, Fe-rich glassy spherules do not contain Mn higher than the EDS detection limit (0.1 wt%; Fig. 5a).

### Transmission electron microscopy (TEM)

**Fig. 6:**
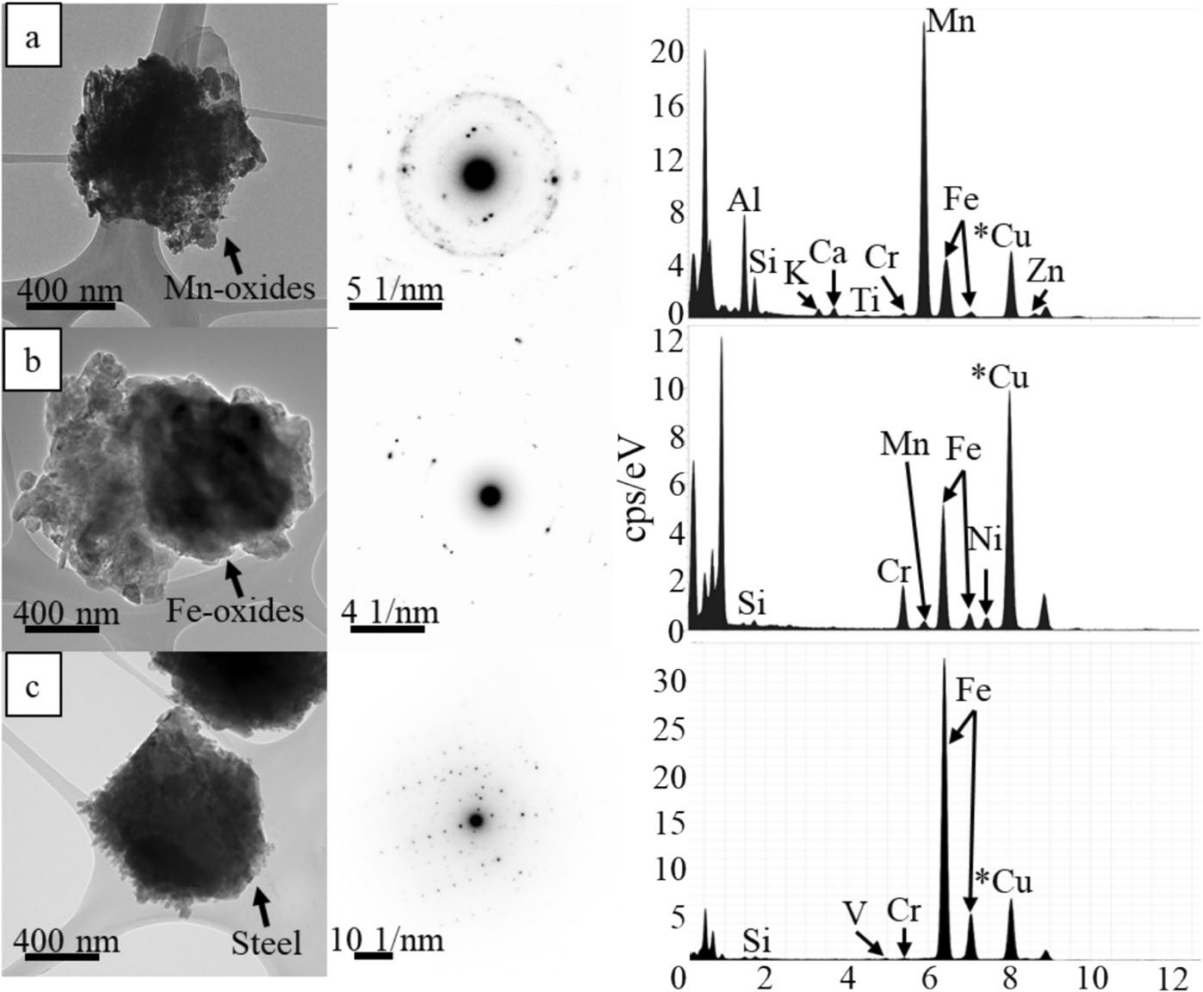
TEM images of a metal-bearing particle and Mn and Fe-oxides identified within Las Vegas road sediment with their respective diffraction patterns and EDXS spectra. Note that the Cu peak is likely due to the use of lacy carbon copper grids for sample analysis. **a**) Mn-oxide aggregate bearing major amounts of Fe, Al, and Si, with Minor Ca, Cr, and Zn present. **b**) Fe-oxide aggregate containing major amounts of Cr and minor Mn, Ni, and Si. **c**) Corroded steel particle rich in Fe and bearing minor Si, V, and Cr

Bright field TEM data indicate that heavy metals, metalloids, and other anthropogenic contaminants are present in Las Vegas road sediment (Fig. 6). In these samples, Fe and Mn-oxides are abundant (Fig. 6a-b), and they are commonly associated with other metals such as Zn, Mn, Cr, and V. Other metal-bearing particles are also identified (Fig. 6c), and are typically enriched in Fe with varying amounts of Ti, Cr, and V present. While Cu may also be present, the use of Cu grids for analysis prohibited the identification of Cu within road sediment in TEM. Despite the ubiquitous presence of anthropogenic spherules in SEM analyses, no spherules were successfully identified using TEM techniques.

### X-Ray fluorescence spectroscopy (XRF)

**Fig. 7:**
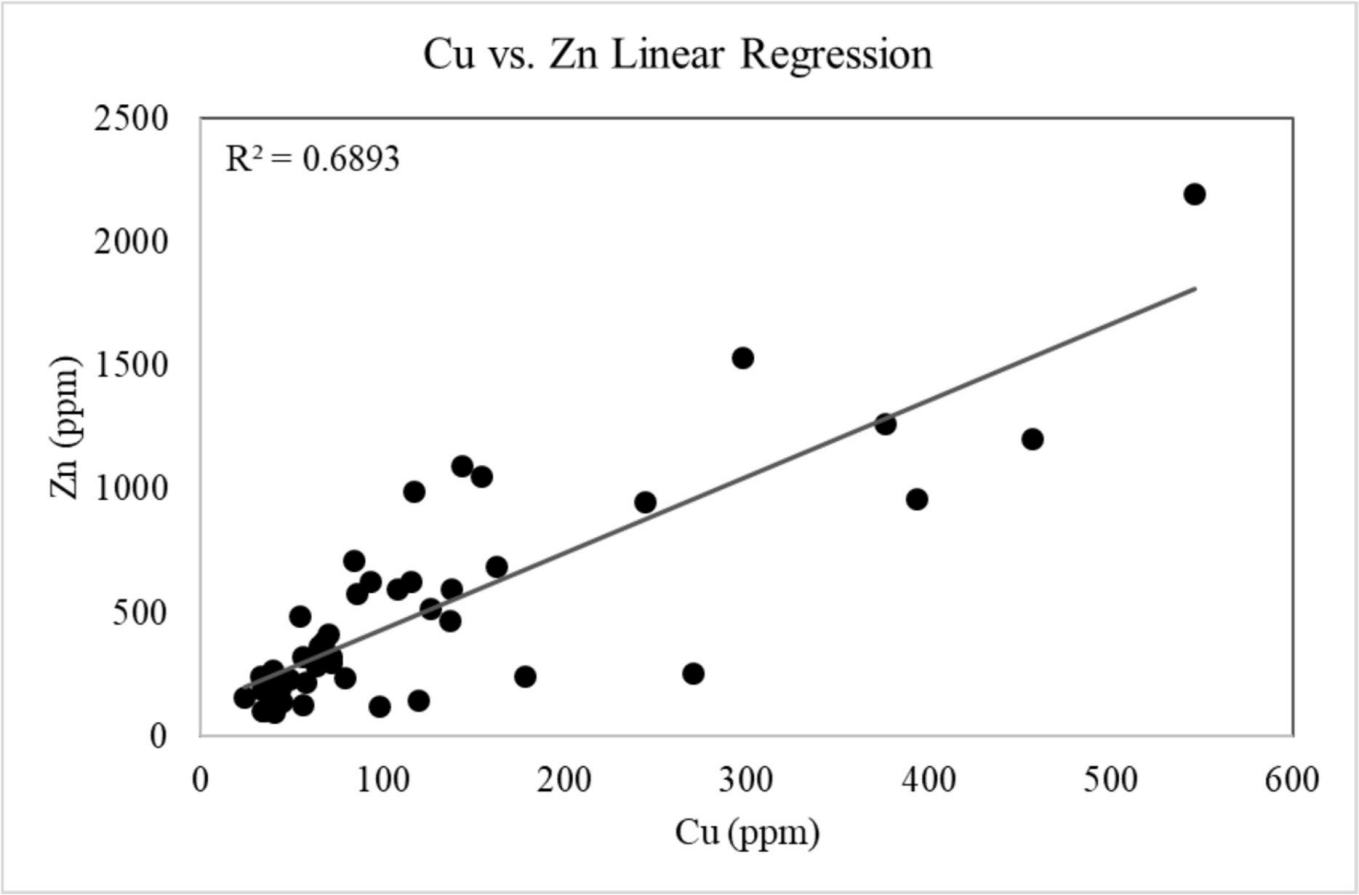
Cu vs. Zn linear regression plot showing a strong positive correlation between the two metals (R^2^ = 0.6893)

**Fig. 8:**
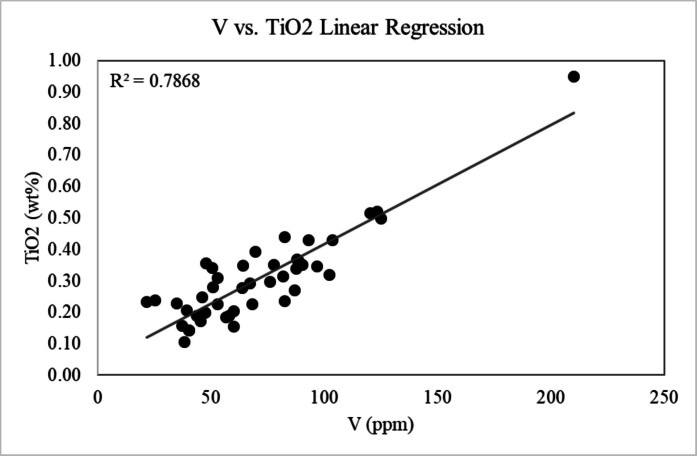
V vs. Ti linear regression plot showing a strong positive correlation between the two metals (R^2^ = 0.7868)

Concentrations of heavy metals and metalloids (e.g., V, Cr, Ni, Cu, Zn, As, Pb, Ti) in Las Vegas road sediment as determined using p-XRF are given in Table 1. Concentrations falling below the instrument detection limit are denoted as BDL. Statistical analysis conducted using a backward step linear regression model shows that a strong positive correlation exists between Zn and Cu (*R*^*2*^ = 0.830) when outliers in the dataset are included, and a moderate positive correlation after outliers are excluded (*R*^*2*^ = 0.566) (Fig. 7). V and Ti show a strong positive correlation both with and without outliers, with Pearson correlation coefficients of 0.887 and 0.801 respectively (Fig. 8).

All road sediment samples analyzed have Cu and Zn concentrations which are higher than regional average background topsoil elemental concentrations as determined by Smith et al. (2013). Concentrations of V, Cr, Ni, As, and Pb in road sediment show more variability in that not all road sediment samples analyzed have concentrations which are higher than those of regional topsoil background (Table 1; Smith et al., 2013).

### Geoaccumulation indices (I_geo_)

**Fig. 9:**
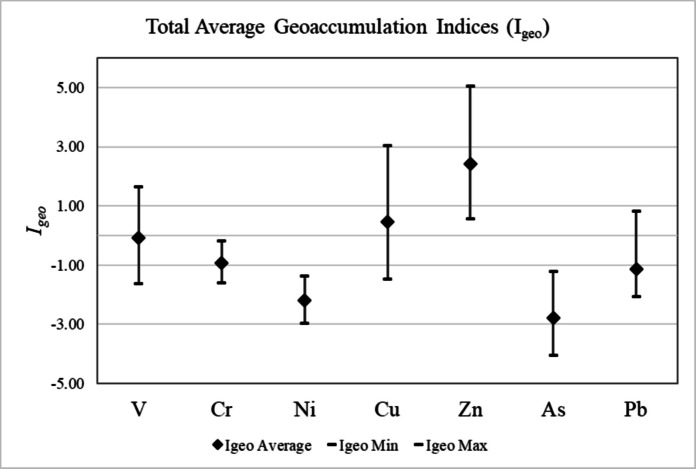
Average *I*_*geo*_values for selected elements across the entire Las Vegas region for all road sediment samples calculated from Smith et al. (2013) topsoil elemental concentrations

**Fig. 10:**
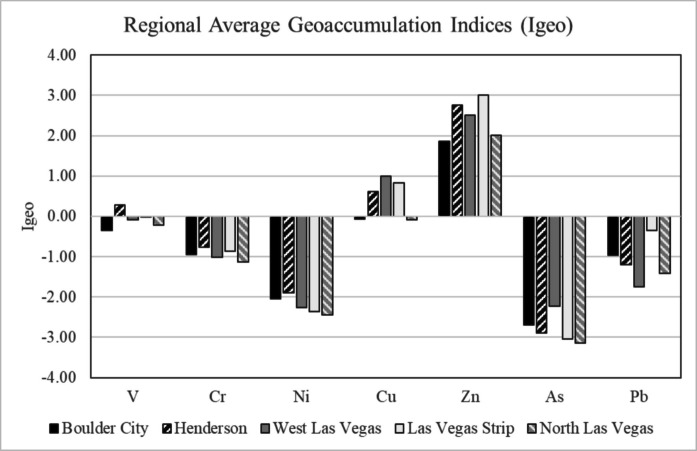
Average *I*_*geo*_ values calculated for each sampling region using Smith et al. (2013) background topsoil concentrations for the Las Vegas area

Geoaccumulation indices (*I*_*geo*_) were calculated using the Müller (1969) method for sampled Las Vegas road sediment using Smith et al. (2013) topsoil elemental concentrations for background elemental concentrations (Figs. 9, and 10, Table 1, 2). Upper continental crust (UCC) elemental concentrations were also utilized for comparative analysis (Rudnick & Gao, 2003; Supplemental Figures, Fig. 1, Tables 3, and 4).

**Table 4:** Average elemental concentrations for the upper continental crust and of geogenic background material from locations near Las Vegas, Nevada

	V^a^	Cr^a^	Ni^a^	Cu^a^	Zn^a^	As^a^	Pb^a^	TiO_2_^b^
Rudnick & Gao (2003) Upper Continental Crust	97	92	47	28	67	4.8	17	0.64
Smith et al. (2013) Nevada Topsoil	44.4	22.5	13.25	13.7	54	6.7	20.1	0.25
Keil et al. (2016) Nellis Dunes CBN 3	62	44	Not reported	32	94	17	23	Not reported
DeWitt et al. (2017) Nellis Dunes CBN 4	100	54	Not reported	37	135	71	34	Not reported
^a^Concentration in ppm; ^b^Concentration in wt%								

Fe_2_O_3_ concentrations were not reported in these studies

Calculated *I*_*geo*_ values for Cr, Ni, As, and Pb for all five areas of Las Vegas are below zero, indicating that these metals are not significantly enriched in road sediments relative to Smith et al. (2013) background concentrations (Figs. 9, and 10; Table 2). Based on the calculated *I*_*geo*_ values, V is also not considered a major pollutant in all areas of Las Vegas, with average *I*_*geo*_ values falling below zero in four of the five sampling areas. The Henderson area is the only location that has an average *I*_*geo*_ value for V above zero (*I*_*geo*_ = 0.27; Fig. 10; Table 2). Calculated *I*_*geo*_ values for Cu indicate that it is enriched in Las Vegas road sediment relative to background concentrations in three of the five sampling locations (Fig. 10; Table 2). The Boulder City and North Las Vegas areas are the only two locations that have average *I*_*geo*_ values for Cu that fall below zero. All other locations (e.g., West Las Vegas, Las Vegas Strip, Henderson) have *I*_*geo*_ values between zero and one (e.g., 0 < *I*_*geo*_ < 1), indicating that these areas are unpolluted to moderately polluted with respect to Cu (Table 2). Four of the five areas in Las Vegas have calculated *I*_*geo*_ values above two (e.g., 2 < *I*_*geo*_ < 3), indicating that Zn is a moderate to strong pollutant. The Boulder City area is the only exception to this, with a calculated *I*_*geo*_ value for Zn between one and two (*I*_*geo*_ = 1.86; Table 2), indicating that Boulder City is moderately polluted.

## Discussion

Analysis via SEM EDS confirms the presence of heavy metals and metalloids such as V, Cr, Ni, Cu, Zn, and Pb in an array of different particles in all road sediment samples from all sampling locations (e.g., metal shavings, spherules; Figs. 2, 3, 4, and 5). However, contaminant types varied widely between each sediment sample analyzed, and while some contaminants (e.g., metal shavings) were identified in some samples, they were not observed in all samples. TEM EDXS analysis further confirms the presence of V, Cr, and Ni in metal-bearing particles. These particles are interpreted to be corroded steel; however, further analyses are needed to confirm this. Mn- and Fe-oxides are observed in samples from all different sampling locations; however, Cu could not be unequivocally identified in particles using this technique due to the contribution of Cu from the sample grids. Similar to observations via SEM EDS, contaminants in each sample determined using TEM EDXS varied in that the same types of contaminants were not observed in all road sediment samples.

Consistent with other road sediment studies (e.g., Flett et al., 2016; LeGalley & Krekeler, 2013; O’Shea et al., 2021a), some Pb-bearing particles identified in this study using SEM EDS are anhedral and appear to form aggregates of multiple smaller particles. Unlike these previous investigations, however, few Pb-bearing particles identified exceeded 20 μm in diameter (Fig. 2a, b, d). Particles containing other metals such as Cu and Zn are generally similar in nature to the Pb-particles identified in that they rarely exceed 10 μm and appear as aggregates of smaller particles (Fig. 2c). When observed using TEM EDXS, Fe and Mn-oxides are prominent and are commonly associated with other metals such as Cr, Mn, Zn, and Ni (Fig. 5). It is possible that Cu was also present in these particles; however, due to the use of Cu grids, identification of Cu within sample material was not possible. Accompanying XRF data has, however, confirmed the presence of Cu in all road sediment samples.

Bulk analysis of all road sediment samples via XRF confirms the variable presence of Cu and Zn. Concentrations of V, Cr, Ni, As, and Pb are also variable, with several road sediment samples exhibiting concentrations of these elements that fall below instrument detection limits (Table 1). Although the presence of these metals has been confirmed in all regions of Las Vegas in certain samples, *I*_*geo*_ analysis indicates that only Zn and Cu should be considered significant pollutants in the greater Las Vegas metropolitan area. These elements have average *I*_*geo*_ values of 0.46 and 2.43 respectively, and all Cu and Zn concentrations as measured by XRF exceed background topsoil concentrations as determined in Smith et al. (2013; Tables 1, and 2, Figs. 9, and 10). Overall, these *I*_*geo*_ values indicate that the region is unpolluted to moderately polluted (0 < *I*_*geo*_ < 1) with respect to Cu, and moderately to strongly polluted (2 < *I*_*geo*_ < 3) with respect to Zn (Table 2, Figs.8, and 9). The designation of these elements as major road sediment pollutants is consistent with classifications in previous studies conducted in the US and other countries (Andrews & Sutherland, 2004; Amato et al., 2009; Apeagyei et al., 2011; Li et al., 2013; Liu et al., 2016; Flett et al., 2016; Men et al., 2018; Dietrich et al., 2018; Dietrich et al., 2019; O’Shea et al., 2020).

Regionally comparative studies such as Keil et al. (2016) and DeWitt et al. (2017), which analyzed two dust samples from Nellis Dunes Recreational Area using inductively coupled plasma mass spectrometry (ICP-MS), report higher concentrations of Cu and Zn in topsoil as compared to Smith et al., (2013; Supplemental Figures, Table 4). However, Cu concentrations in road sediment still exceed those reported by Keil et al. (2016) in 98% of samples and by DeWitt et al. (2017) in 89% of road sediment samples analyzed. Zn shows a similar trend, with concentrations exceeding those reported by Keil et al. (2016) in 100% of samples and by DeWitt et al. (2017) in 85% of samples. Winds in the Nellis Dunes area typically blow from the northeast between the months of November and March and from the south through the months of April to September (Goossens & Buck, 2009), and thus it is possible that dust from Nellis Dunes contributes to Zn and Cu concentrations in Las Vegas road sediment. Despite being a potential contributor of Zn and Cu to the Las Vegas area, the recreational area is not likely the primary source of these elements in road sediment (DeWitt et al., 2017; Keil et al., 2016).

Overall, results from this work show that Zn is the most abundant metal pollutant in Las Vegas road sediment followed by Cu, with all Zn and Cu concentrations in road sediment exceeding the average concentration of these metals in regional topsoil (Smith et al., 2013). Anthropogenic sources of Zn in urban areas are typically traced back to vehicular activity, and existing literature has commonly attributed Zn and Cu enrichment to tire and brake wear, with Zn also being attributed to motor oil (Davis et al., 2001; Andrews & Sutherland, 2004; Irvine et al., 2009; Apeagyei et al., 2010; Li et al., 2013; Hwang et al., 2016).

Consideration of the coefficients of determination (*R*^2^) confirms that Zn and Cu have a moderate (*R*^*2*^ = 0.320 excluding outliers) to strong positive correlation (*R*^*2*^ = 0.641 with outliers included; Fig. 7a). Zn and Cu also record the highest average *I*_*geo*_ values overall; thus, this correlation is interpreted as demonstrating a common point of origin for these two metals (e.g., vehicular activity; Andrews & Sutherland, 2004; Wiseman et al., 2021). Vehicular activity may not be the only contributor of Cu and Zn in road sediment, and other potential sources of these elements should be considered. For example, the Three Kids Mine, located approximately 4 km NE of Henderson and 11 km NW of Boulder City, may contribute Zn as well as other metals and metalloids (i.e., Cu, As, Pb) to road sediment in these localities more so than other surrounding Las Vegas regions. Park et al. (2014) documented that mine tailings at the Three Kids Mine had concentrations of Cu (260 ppm) and Zn (2050 ppm) exceeding background concentrations of < 50 ppm and 69 ppm, respectively. Although wind directions typically come from the southwest (away from Henderson and Boulder City), this abandoned mine site cannot be discounted as a source of these metals in the Henderson region (Park et al., 2014).

Other anthropogenic sources of Cu and Zn may include steel, brass, and bronze manufacturing, paints, ceramics, etc. (ATSDR, 2005; ATSDR, 2022). Initial identification of Zn-bearing particles via SEM and TEM has shown that Zn tends to occur with other metals such as Mn and Fe as well as Cu (Fig. 2c). Fe and Mn-oxides are known to occur together in the environment and often undergo redox cycling, which may cause changes in the valence states of Fe and Mn (Li et al., 2024; Liu et al., 2022). Although the oxidation states of these elements are unknown in this study, Zn may occur as a constituent of the Fe- and Mn-oxides or other clay minerals identified in the region (i.e. sepiolite, palygorskite), as Zn has the potential to adsorb to the surface of these minerals over a wide range of environmental conditions (Parker et al., 1978; García-Sánchez et al., 1999; Keil et al., 2016; Komárek et al., 2018; Li et al., 2024). Cu-bearing particles identified using SEM may occur with a wide range of metals such as Pb, Ti, Mn, and Fe; therefore, suggesting that Cu and Zn do not always occur together and are thus not only derived from vehicular activity (Fig. 2a, c).

Other heavy metals such as V, Cr, Ni, and Pb, along with the metalloid As, were detected using SEM and TEM analyses. Long-term or acute exposure to any of these metals may cause deleterious health effects in humans, and enrichment of these metals in road sediment may be cause for concern.

Though rarely identified using SEM, V is confirmed to be present in all but three road sediment samples analyzed by XRF (Table 1), and V concentrations are found to be higher than regional topsoil elemental concentrations (Smith et al., 2013) in 36 samples analyzed. V was also rarely observed using TEM EDXS techniques, and where observed, occurs with other metals such as Fe and Cr (Fig. 6c). Overall, the lack of observable V-bearing particles using SEM and TEM may be due to V being present in concentrations lower than the detection limits for both instruments. Statistical analysis shows that V and Ti have a strong positive correlation both with outliers included (*R*^*2*^ = 0.787) and excluded (*R*^*2*^ = 0.642) from the dataset (Fig. 8). Average V and Ti concentrations are higher in Henderson in comparison to all other sampling regions, and this may be due to Ti alloy manufacturing which is prevalent in the region (TIMET, 2024). Other sources of V in the environment may include vehicles or ceramics (ATSDR, 2012). Geogenic Fe-V-Ti oxides are assessed to not be very likely or major contributors to the TiO_2_-V trend because of a lack of correlation (*R*^*2*^ = 0.08) of V and TiO_2_ with Fe_2_O_3_ concentrations. Ti and petroleum material in the road system are independent, and thus V and Ti concentrations in road sediment conservatively appear to be influenced by anthropogenic alloys.

While Cr may be derived from natural sources in and around Las Vegas (DeWitt et al., 2017; Keil et al., 2016, 2018), it may also be derived from anthropogenic sources such as industrial activity and paints (ATSDR, 2012; Dietrich et al., 2022b). Cr occurs primarily as Cr(0), Cr(III), or Cr(VI), and although small amounts of Cr(III) are essential for human health, exposure to Cr(VI), a well-known carcinogen, may result in negative health effects (ATSDR, 2012). The oxidation state of Cr in Las Vegas road sediment is unknown; however, Cr(VI) may be derived from sources such as stainless steel, chrome plating, yellow road paint, or other ferrochromium alloys (ATSDR, 2012; White et al., 2014; Dietrich et al., 2022b). Several metal shavings and steel particles identified using SEM and TEM in this study contained Cr. The valence state of this Cr cannot be determined through SEM, TEM, or XRF analyses; however, Cr(0) is typically used to aid in the corrosion resistance of steel, and when corrosion does occur, Cr(III) is the primary ion released into the environment, which is not considered a human health hazard (British Stainless Steel Association, 2024). Despite this, Cr(III) may be converted to Cr(VI) during heating in the presence of oxygen (Apte et al., 2006). Cr(VI) may also enter the environment through the degradation of PbCrO_4_-based yellow road paint, which has been shown to have been used in various locations in the US (e.g., OH, PA) (Dietrich et al., 2022a, 2022b; Flett et al., 2016; O’Shea et al., 2021a, 2021b; White et al., 2014). PbCrO_4_-based road paint has a poorly constrained history of use, and no studies have been completed on the presence of this paint in Las Vegas; thus, further investigation of yellow road paint in Las Vegas is warranted in order to better identify sources of Cr(VI) and Pb in the environment.

Pb-bearing particles are interpreted as being elemental Pb due to similarities in textural characteristics observed in other studies that examine road sediment (Dietrich et al., 2019; Flett et al., 2016; LeGalley & Krekeler, 2013). Dietrich et al. (2022a) demonstrated that in road sediment studies completed after 1990 in the US, Pb concentrations decreased significantly, which is likely due to the ban of leaded gasoline in automobiles. However, Pb is known as a “legacy pollutant”, meaning that it takes many years to break down and will remain present in the environment for decades. Thus, leaded gasoline could be a potential source for the elemental Pb present in the road sediment. Nevada is also one of nine US states that has not banned leaded tire weights; thus, these may also be a possible source of elemental Pb present in the Las Vegas region (Ayuso & Foley, 2020; Hwang et al., 2016). Overall, traffic density may affect the presence of elemental Pb, as previous studies have noted an increase in heavy metal pollution of road sediment in high traffic areas (Andrews & Sutherland, 2004; Apeagyei et al., 2011).

Although Pb is present in Las Vegas road sediment, bulk concentrations are lower than those reported in other recent studies of road sediment in the US (e.g., Dietrich et al., 2022b; LeGalley & Krekeler, 2013; O’Shea et al., 2021a, 2021b). One potential explanation for this could be that these recent studies have all been conducted in areas of the US (e.g., Ohio, Pennsylvania, and Indiana) which have had an extensive history of industrial activity such as coal-burning power plants and metal manufacturing (LeGalley et al., 2013; Flett et al., 2016; Dietrich et al., 2018; O’Shea et al., 2021a; Allen et al., 2024).

In addition to Cr and Pb in Las Vegas road sediment, the presence of As in road sediment may be attributed to the presence of Mn- and Fe-oxide/oxyhydroxide minerals, which are known to adsorb As (Soukup et al., 2012). Dusts from the Nellis Dunes Recreational Area, located 15 miles northeast from the center of Las Vegas, may also contribute to As enrichment in road sediment, as previous literature has shown elevated levels of As in dusts from Nellis Dunes as compared to regional background topsoil concentrations (DeWitt et al., 2017; Goossens et al., 2015; Keil et al., 2016; Soukup et al., 2012). Smith et al. (2013) found that average As concentrations in topsoil (~ 77 ppm) from around the greater Las Vegas region are lower than concentrations noted in Goossens et al. (2015) (< 10 ppm up to ~ 7058 ppm). In 36 out of 46 road sediment samples analyzed in this study, As concentrations exceeded the Smith et al. (2013) average background As concentrations, but none exceeded the upper concentration limit as determined by Goossens et al. (2015). Therefore, while some As may be attributed to dust derived from Nellis Dunes, it does not appear to be a major source of As as a contaminant in road sediment in this study. Another source of As in the region may be attributed to salts and acid rock drainage from decorative water-efficient landscaping rock containing an abundance of sulfide minerals (Mrozek et al., 2006). Mrozek et al. (2006) found that in the Las Vegas region where this landscaping rock was utilized, concentrations of As, Cu, Mo, Pb, and Cr were higher than regional background concentrations in the salt crusts and soil surrounding these landscaping rocks. Though this region receives little annual precipitation, precipitation or irrigation in areas where these rocks are present may cause these salts to dissolve, thus releasing these metals into the environment, or acid rock drainage may occur due to the oxidation of sulfide minerals within these rocks (Mrozek et al., 2006). Although there may be several sources of As in the Las Vegas region that may contribute to road sediment, As is not considered to be a pollutant within the areas of Las Vegas sampled, as calculated *I*_*geo*_ values for As did not exceed an *I*_*geo*_ of 0 for any sample taken.

In Las Vegas road sediment, metal shavings were imaged using SEM BSD (Fig. 3); however, these shavings contained no detectable Zn when analyzed via EDS. Instead, Zn most often occurred in anhedral particles < 2 µm in diameter, making these particles respirable and thus possibly harmful to humans if inhaled (Wiseman et al., 2018; Wiseman & Zereini, 2014; Padoan et al., 2017; Khan & Strand, 2018; Wiseman et al., 2021; Dietrich et al., 2022a; US EPA, 2024). Zinc was also observed to occur with Fe-oxides/oxyhydroxides. This is consistent with other studies which concluded that Zn is often deposited as fine particulate matter which dissolves and then preferentially adsorbs onto Fe-oxides/oxyhydroxides (Soukup et al., 2012).

Anthropogenic spherules were identified in all Las Vegas road sediment samples analyzed via SEM; however, no spherules were successfully identified using TEM imaging techniques. Spherules are a well-recognized contaminant in the environment and have been documented in other road sediment studies (Allen et al., 2024; Dietrich et al., 2018, 2019, 2022a, 2022b; LeGalley & Krekeler, 2013; Liu et al., 2016; Magiera et al., 2011, 2013). Many of the technogenic spherules identified in the Las Vegas road sediment samples had textures consistent with mechanical abrasion and weathering similar to those observed in LeGalley and Krekeler (2013) and Allen et al. (2024). Although the exact source of anthropogenic spherules in Las Vegas road sediment is unknown, previous literature has indicated that these spherules are most often derived from combustion processes (e.g., coal burning, smelting processes) (Allen et al., 2024; Dietrich et al., 2019; Ismail et al., 2007; Kutchko & Kim, 2006; LeGalley & Krekeler, 2013; Magiera et al., 2011, 2013). The spherules identified in this study are comparable in texture and chemical composition to those found in other road sediment studies in the Eastern and Midwestern US (Allen et al., 2024; Dietrich et al., 2019; Flett et al., 2016; LeGalley & Krekeler, 2013) as well as other fly ash characterization studies (Kutchko & Kim, 2006; Ismail et al., 2007; Magiera et al., 2011, 2013).

In studies of road sediment from the Midwestern and Eastern US, anthropogenic spherules often occurred with heavy metals such as Pb and were commonly observed in areas where coal burning and industrial activity have been historically prevalent. In Las Vegas, however, these spherules were not closely associated with elevated Pb concentrations, and this region does not have an extensive history of industrial activity. Thus, these anthropogenic spherules may be attributed to different sources. Some Si-rich glassy spherules may still be derived from industrial activities in the region; however, other sources such as vehicle exhaust emissions as well as building and road paints may be other major contributors (White et al., 2014; Shetye et al., 2019). Though metal-rich spherules are typically derived from manufacturing processes, welding and similar activities can yield anthropogenic spherules with similar compositions and dendritic and skeletal surface textures (Brożek-Mucha, 2015).

## Conclusions and implications

Relatively few road sediment studies have been conducted in the conterminous US, and none have thus far been conducted in arid to semi-arid environments. This study applied a multianalytical approach to investigate the contaminants present in Las Vegas road sediment to better understand the potential environmental health risks. Pb, Zn, and Cu are the elements of highest concern; however, Pb concentrations are notably lower than those in other communities in more temperate climates (i.e., Hamilton and Middletown, OH, Gary, IN, Philadelphia, PA). Zn and Cu are the most abundant pollutants in Las Vegas road sediment and are positively correlated, thus suggesting a common origin: these metals are closely associated with vehicular activity. The presence of anthropogenic spherules in Las Vegas road sediment, combined with low Pb concentrations as compared with Midwestern and Eastern US studies, suggests that coal burning and industrial activity may not be a major contributor to road sediment pollution in the region. Instead, these spherules may be primarily derived from sources such as vehicle exhaust emissions, paints, welding, etc.

Overall, this study indicates significant variation and differences in the chemical and morphological nature of contaminants and geogenic material in road sediment between climate zones. Our findings suggest that although contaminant (e.g., metal shavings) and pollutant types (e.g., Zn and Cu) in road sediment from an arid environment like Las Vegas may be similar to those found in road sediment from more humid climates (e.g., Ohio, Hawaii, Pennsylvania), sources may vary significantly, and environmental processes such as precipitation or lack thereof may affect the ability of certain contaminants to mobilize in these environments. For example, arid environments may see more wind transport of dust or lack of adsorption/desorption to other minerals occurring due to lack of precipitation. This investigation supports the new directions of research recommended in Dietrich et al. (2022a) and will add to the body of knowledge regarding road sediment in the US. Additional analyses such as bulk sample chemical characterization via inductively coupled plasma mass spectroscopy (ICP-MS) and isotopic analysis using Pb, Zn, and Cu isotopes are needed to constrain potential sources for much of the metal pollutants present in the Las Vegas region.

## Supplementary Information

Below is the link to the electronic supplementary material.

ESM 1 (DOCX 38.9)

## Data Availability

No datasets were generated or analysed during the current study.
