# Supplementary material for: The nature and distribution of road sediment contaminants in the greater Las Vegas, Nevada area
Source: Environ Monit Assess. 2025 Dec 3;198(1):5. doi: 10.1007/s10661-025-14725-9 (PMC12675632; doi:10.1007/s10661-025-14725-9)
Supplement: Supplementary file 1 — (DOCX 38.9) [file 10661_2025_14725_MOESM1_ESM.docx]

# **Supplemental Figures**

**
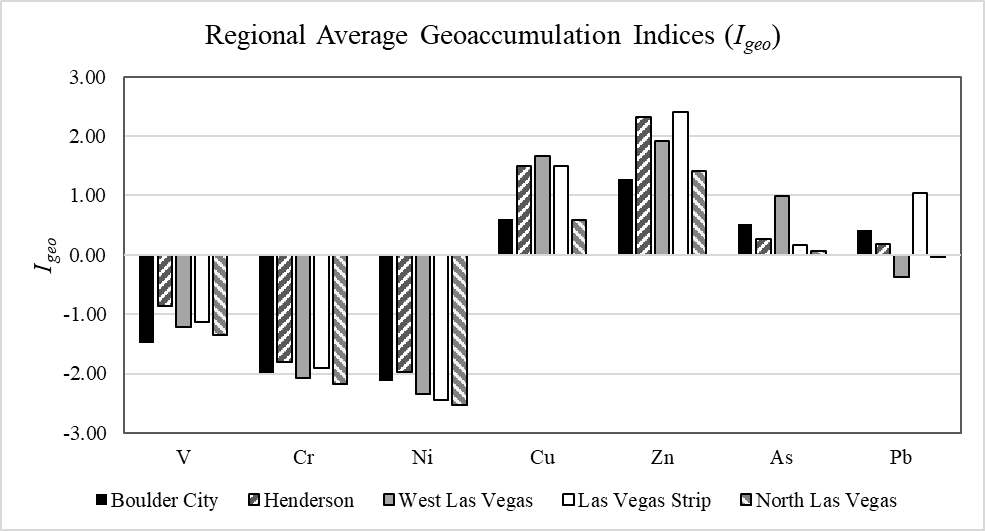
**

**Fig. 1** Average *I_geo_* values calculated for each sampling region using Rudnick & Gao (2003) background topsoil concentrations for the Las Vegas area.
